# Supplementary material for: Injury-activated glial cells promote wound healing of the adult skin in mice
Source: Nat Commun. 2018 Jan 16;9:236. doi: 10.1038/s41467-017-01488-2 (PMC5770460; doi:10.1038/s41467-017-01488-2)
Supplement: Supplementary file 1 — Supplementary Information [file 41467_2017_1488_MOESM1_ESM.pdf]

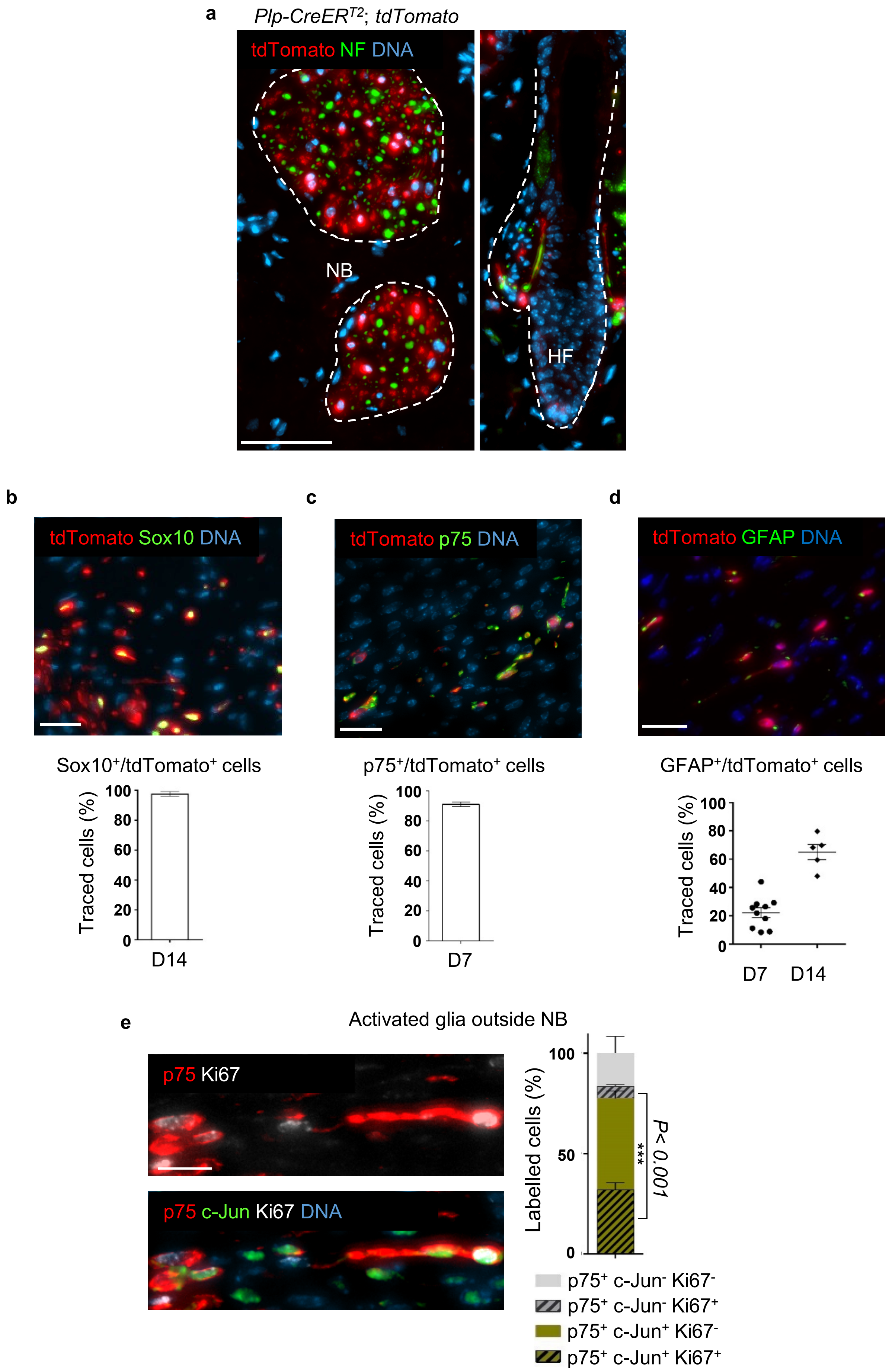


Supplementary Figure 1

**Supplementary Figure 1** *Plp-CreER*$^{T2}$ lineage tracing in intact and injured skin. **a** tdTomato reporter expression in nerve bundles (NB) and around hair follicles (HF). **b** Immunofluorescence staining of *Plp-CreER*$^{T2}$ traced cells in granulation tissue for tdTomato (red) and Sox10$^{+}$ (green) and quantification of the percentage of Sox10$^{+}$ per tdTomato$^{+}$ cells in the granulation tissue of injured skin at D14 (N=2, n=6). **c** Immunofluorescence staining of tdTomato$^{+}$ *Plp-CreER*$^{T2}$-traced cells in granulation tissue (red) with p75$^{+}$ (green) and quantification of the percentage of p75$^{+}$ per tdTomato$^{+}$ cells in the granulation tissue of injured skin at D7 (N=4, n=10). **d** Immunofluorescence staining of D14 tdTomato$^{+}$ *Plp-CreER*$^{T2}$ traced cells (red) with glial fibrillary acidic protein (GFAP) (green) and quantification of the percentage of GFAP$^{+}$ per tdTomato$^{+}$ cells in the granulation tissue of injured skin at D7 (N=3, n=10) and D14 (N=2, n=6). **e** Immunofluorescence and corresponding quantification of proliferative (Ki67$^{+}$), activated (c-Jun$^{+}$), de-differentiated (p75$^{+}$) glial cells outside NBs (N=6, n=9). Data are represented as mean±SEM. Scale bars, 50μm (**a**), 25μm (**b**,**c**,**d**).

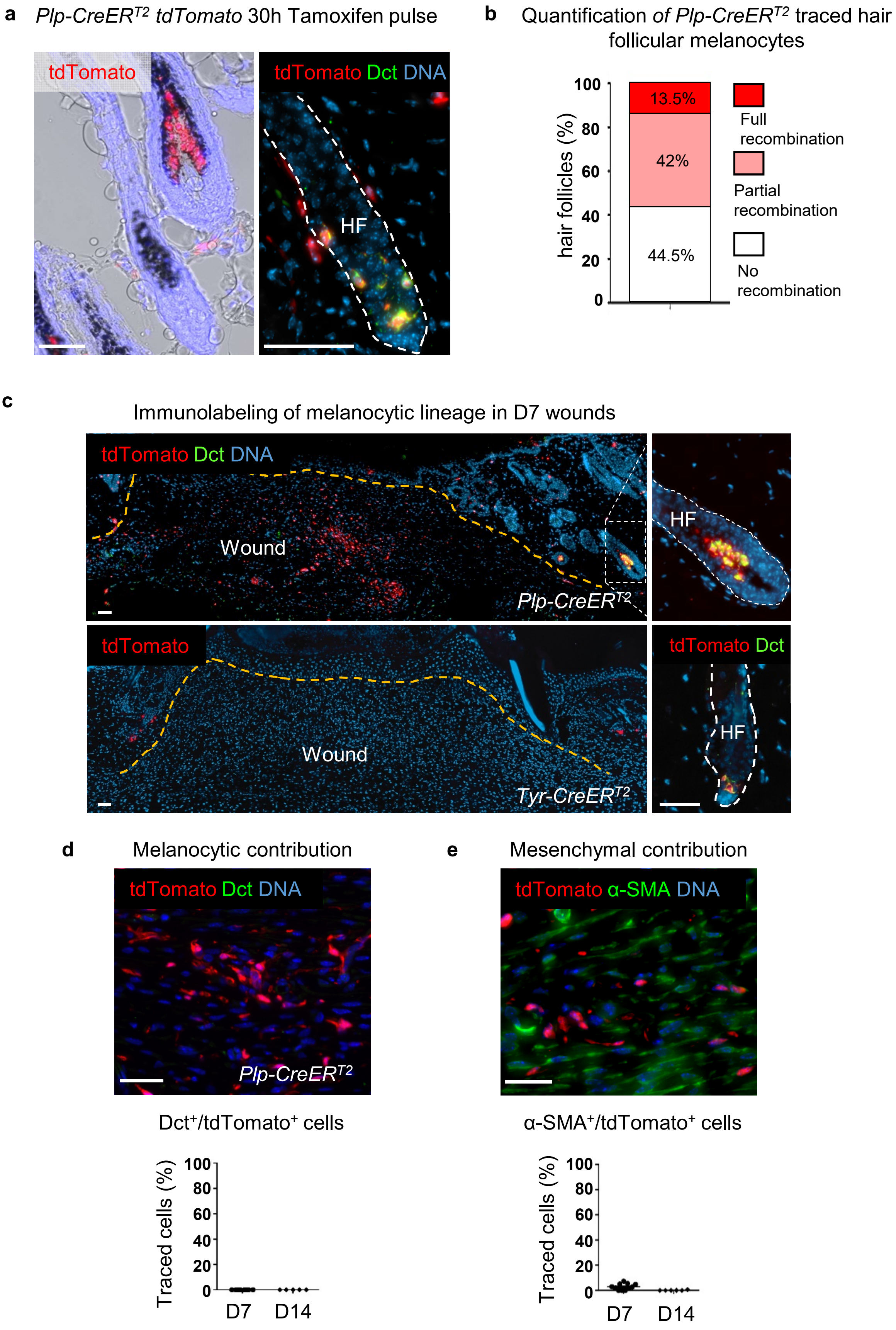


Supplementary Figure 2

**Supplementary Figure 2** *Plp-CreER$^{T2}$*-mediated recombination of adult melanocytes in anagen hair follicles and absence of melanocytic lineage contribution to the wound bed of injured skin. **a** *Plp-CreER$^{T2}$* driven recombination of anagen murine skin shows only partial tdTomato reporter expression in hair follicles (HFs), 30h post TM injection. Co-labelling of tdTomato (red) and the melanocytic marker Dopachrome tautomerase Dct (green). **b** Quantification of percentage of HFs displaying no, partial or complete *Plp-CreER$^{T2}$* mediated recombination of their respective melanocytic population, 30h post-TM injection (n=220 HFs, N=1). **c** Immunolabelling for tdTomato reporter (red) and Dct (green) of *Plp-CreER$^{T2}$* and *Tyr-CreER$^{T2}$* traced melanocytic cells in D7 wound bed and in an adjacent HF fails to reveal traced melanocytes in the granulation tissue upon injury. Sections were counter stained with Hoechst 33258 (blue). **d** Immunofluorescence staining of tdTomato$^{+}$ *Plp-CreER$^{T2}$* traced cells (red) in granulation tissue with melanocyte marker Dopachrome tautomerase (Dct) (green) and quantification of the percentage of Dct$^{+}$ per tdTomato$^{+}$ cells in the granulation tissue of injured skin at D7 (N=3, n=8) and D14 (N=2, n=5). **e** Immunofluorescence staining of tdTomato$^{+}$ *Plp-CreER$^{T2}$* traced cells (red) with myofibroblast marker alpha smooth muscle actin (α-SMA) (green) and quantification of the percentage of α-SMA$^{+}$ per tdTomato$^{+}$ cells in the granulation tissue of injured skin at D7 (N=3 animals, n=11 wounds) and D14 (N=2, n=6). Scale bars, 50µm.

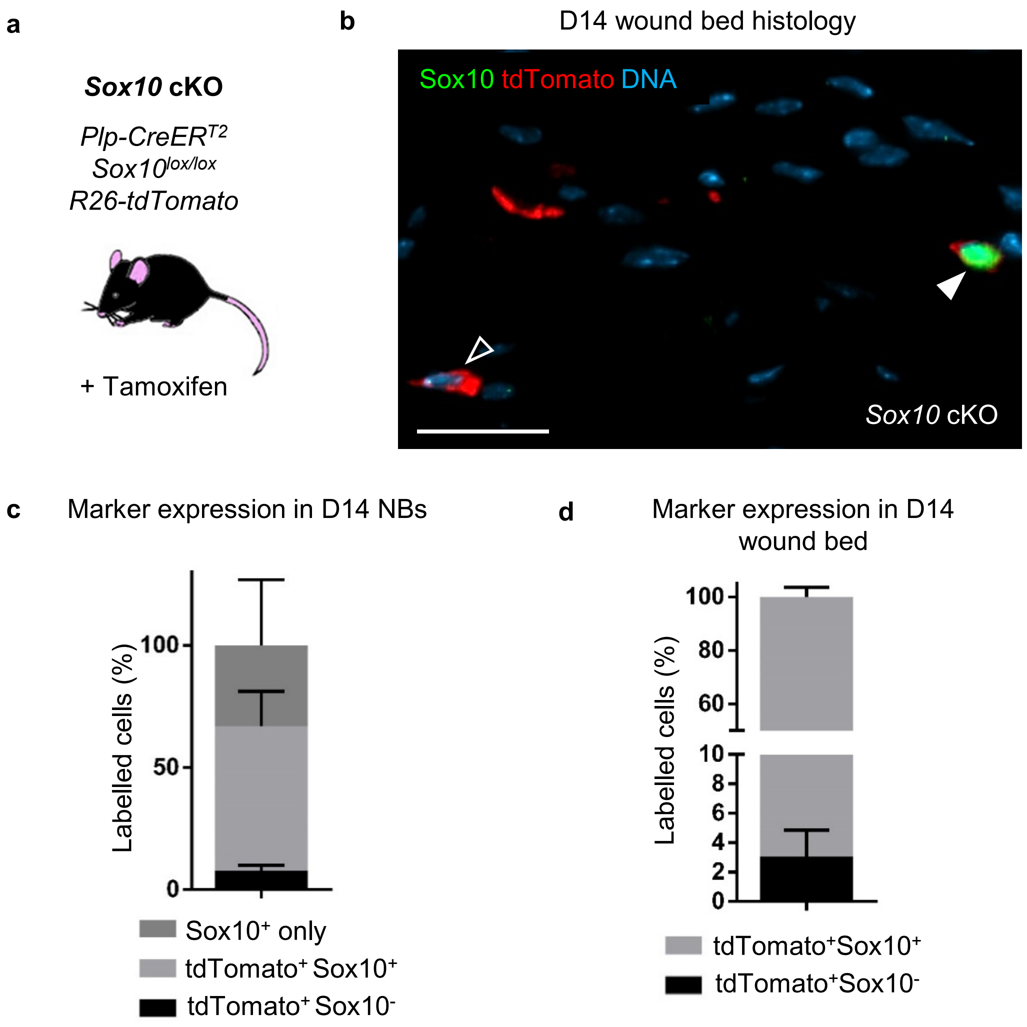


Supplementary Figure 3

**Supplementary Figure 3** Fate of *Plp-CreER*$^{T2}$-traced cells after *Sox10* cKO. **a** Schematic of the murine model used to trace glial cells in skin of *Sox10* cKO mice. **b** Immunolabelling of Sox10 (green) and tdTomato tracer (red) of D14 *Sox10* cKO wound bed. Black arrowhead, tdTomato$^{+}$ Sox10$^{-}$ cell; white arrowhead, tdTomato$^{+}$ Sox10$^{+}$ cell **c**,**d** Corresponding quantification of Sox10 expression among *Plp-CreER*$^{T2}$-traced tdTomato$^{+}$ cells (**c**) within nerve bundles (NBs) and (**d**) in the wound bed (N=2 animals, n=4 wounds for each condition). Scale bar, 20μm.

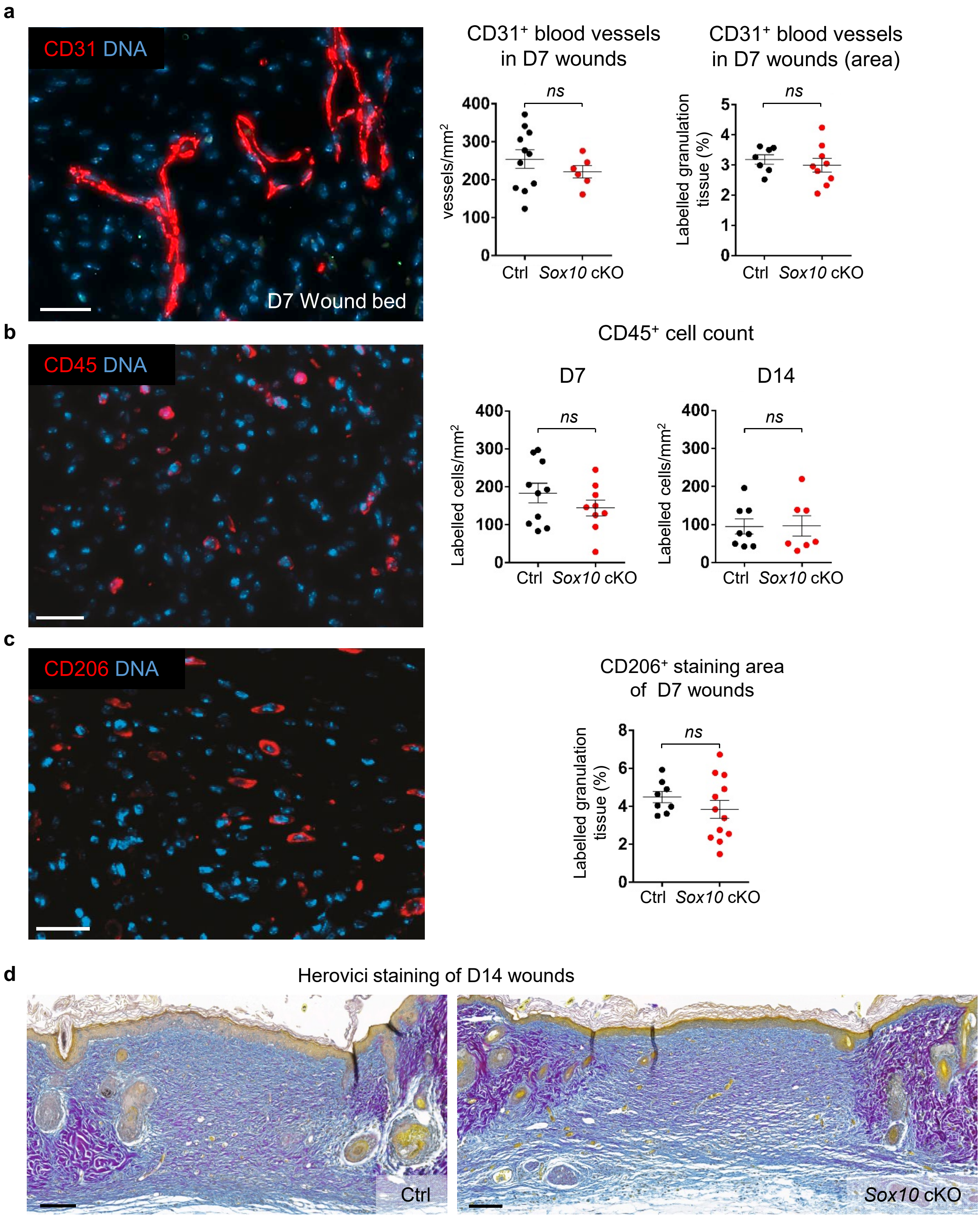


Supplementary Figure 4

**Supplementary Figure 4** Neovascularization, immune cell infiltration and collagen maturation are not affected upon loss of glial contribution to tissue repair. **a** Immunolabelling of CD31 (red) in granulation tissue at D7 and related quantification of number of blood vessels $mm^{-2}$ and percentage of $CD31^{+}$ area per total granulation tissue surface in skin wounds at D7 did not display noticeable differences upon loss of *Sox10*. **b** Immunolabelling of CD45 (red) in granulation tissue at D7 and quantification of $CD45^{+}$ cells in the skin wounds at D7 and D14 did not show any difference upon *Sox10* depletion. **c** Immunolabelling of CD206 (red) in granulation tissue at D7 and quantification of $CD206^{+}$ area in skin wounds at D7 showed no significant changes upon *Sox10* cKO. **d** Herovici staining for collagen maturation performed on D14 sections of Ctrl and *Sox10* cKO wounds. Data are represented as mean±SEM of N=3, n=11 (Ctrl), N=2, n=6 (*Sox10* cKO) (**a** – blood vessels $mm^{-2}$); N=3, n=7 (Ctrl), N=6, n=9 (*Sox10* cKO) (**a** – blood vessel area); N=3, n=10 (Ctrl D7), N=5, n=8 (Ctrl D14); N=5, n=9 (*Sox10* cKO D7), N=4, n=7 (*Sox10* cKO D14) (**b**); N=5, n=8 (Ctrl), N=8, n=12 (*Sox10* cKO) (**c**) Scale bars, 25µm (**a**-**c**), 100µm (**d**).

**a** Western blot analysis of α-SMA in total D7 wound protein

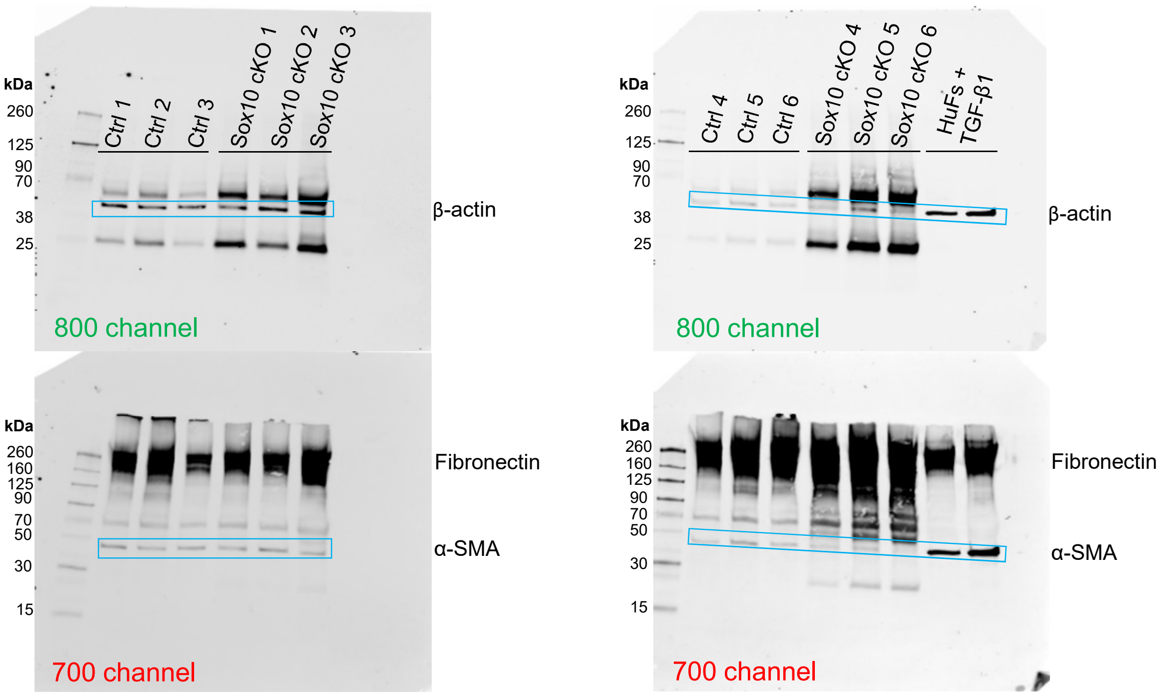


**b** Western blot analysis of Periostin in total D7 wound protein

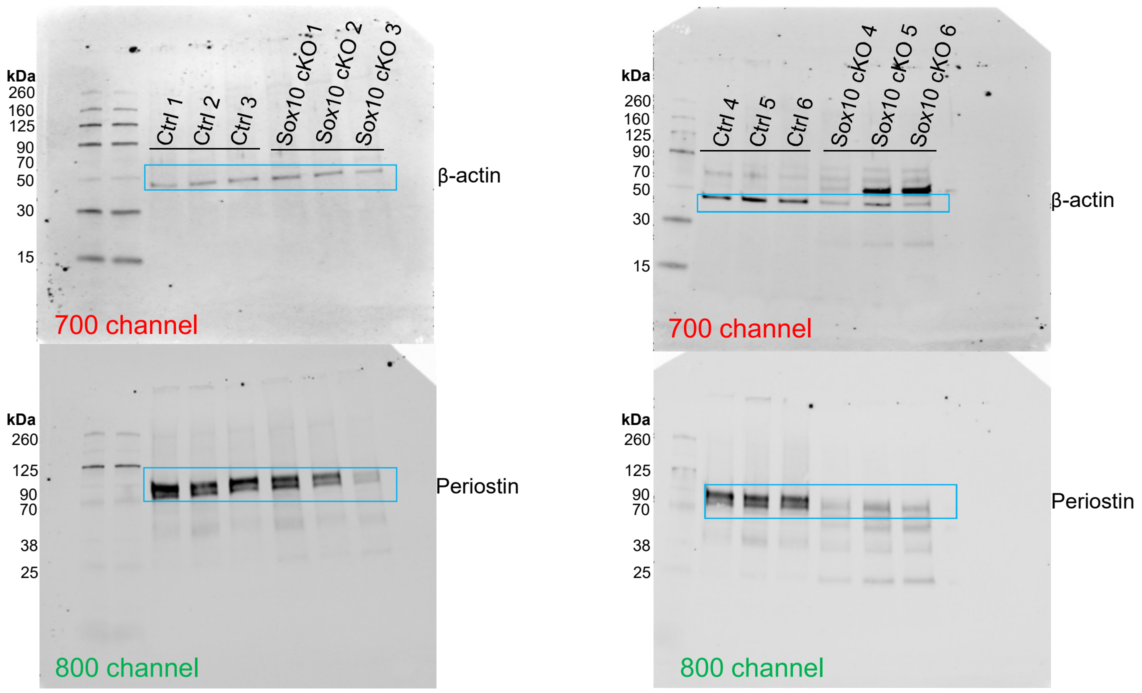


Supplementary Figure 5

**Supplementary Figure 5** Full scans of western blots performed on lysed wound samples. **a** Full scans of α-SMA western blots corresponding to quantification in Figure 5j (N=3, n=6) Human fibroblasts treated with TGF-β1 were used as control. **b** Full scans of Periostin western blots corresponding to quantification in Figure 5k (N=3, n=6) Samples were standardized to β-actin expression.

**Supplementary Table 1** Top upregulated growth factor and cell signalling related genes with reported functional relevance.

| Gene name | Log2 FC | Reported function |
|---|---|---|
| Tnn | 3.007 | Tenascin-W mediates neurite repulsion from hippocampal explants[1] and has anti-adhesion matrix modulating properties[2,3]. |
| Areg | 2.826 | EGFR ligand amphiregulin is a growth factor for keratinocytes important in wound healing[4,5]. TGFβ regulates amphiregulin to promote lung fibrosis[6]. Important in inflammatory wound response[7]. |
| Tnc | 2.742 | Tenascin-C loss leads to corneal wound healing delay and decreased myofibroblast generation[8] and regulates myofibroblast recruitment after cardiac injury[9]. It is strongly expressed by Schwann cells during Wallerian degeneration[10]. |
| Wisp1 | 2.546 | Role in wound healing suggested[11]. |
| Inhba | 2.477 | Subunit of activin – a crucial factor in wound healing[12]. Expressed in the granulation tissue[13]. |
| Aspn | 2.476 | Anti-fibrotic. Found after spinal cord injury[14] Inhibits TGF-β/SMAD signalling in osteoarthritis settings[15,16]. |
| Tnfsf14 | 2.309 | LIGHT plays role in the resolution of inflammation in wound healing[17], promotes collagen deposition and epidermal thickening and fibrosis in mouse skin[18]. It has been shown to be upregulated at the site of regeneration following nerve injury, but not reported in Schwann cells[19,20]. |
| Btc | 2.306 | EGF family protein that regulates new hair follicle formation, hair cycle induction and angiogenesis after wounding[21]. Induces Schwann cell migration[22]. |
| Fgf5 | 2.173 | FGF5 expression is increased upon injury[23]. Promotes angiogenesis [24] and regulates hair growth cycle[25]. Found to be upregulated in the Schwann cells of the injured nerve[26]. |
| Epha3 | 2.162 | EphaA3 receptor is upregulated upon spinal cord injury [27]. |
| Ltbp2 | 2.092 | As an inhibitor of FGF-2 may modulate wound repair and contribute to fibrotic diseases[28] |
| Hilpda | 1.965 | Induced by hypoxia and HIF1[29], an important factor in wound healing[30]. |
| Cd38 | 1.912 | CD38 is found on several cell types in the brain including astrocytes and facilitates recovery from the brain injury[31]. |
| Cpz | 1.911 | Carboxypeptidase Z (CPZ) – ECM protein that modulates Wnt signalling[32] Function in wound healing has not been reported so far. |
| Trf | 1.897 | Provided by PNS and necessary in amphibian limb regeneration blastemas[33]. Expressed in Schwann cells after injury[34]. |

| | | |
|---|---|---|
| Pdgfb | 1.889 | PDGF is important in wound healing[35] Topical application of PDGF BB enhances wound healing[36], however, conflicting data on the effects of PDGF BB exists[37]. |
| Metrn | 1.868 | Neurotrophic factor Meteorin regulates glial cell differentiation and promotes axonal extension[38] and promotes gliogenesis after striatal injury[39]. |
| Bgn | 1.792 | Biglycan is an ECM proteoglycan able to induce proto-myofibroblast phenotype and migration of fibroblasts[40]. |
| Hgf | 1.746 | HGF promotes wound healing[41–43] and is implicated in PNS development and injury repair[44,45]. |
| Lgmn | 1.707 | Legumain is essential for recovery after spinal cord injury in zebra fish[46] and determines bone marrow stem cell differentiation[47]. |
| Gfra1 | 1.700 | Found on Schwann cell membranes and can be secreted[48]. Important for injured motor neuron fate determination[49]. |
| Igf1 | 1.646 | IGF1 is important for keratinocyte migration and wound re-epithelialization[50]. Promotes wound healing in oestrogen-deprived mice[51]. Can be produced by Schwann cells upon injury[52] and facilitates nerve regeneration[53]. |

**Supplementary Table 2** Top upregulated TGF-β signalling related genes with reported functional relevance.

| Gene name | Log2 FC | Reported function |
|---|---|---|
| Postn | 3.044 | Activates TGF-β pathway[54]. Promotes wound healing[55,56] and myofibroblast differentiation in the wound[57]. Important for wound melanoma metastasis[58] and Schwann cell migration[59]. Astroglial periostin promotes axonal regeneration after spinal cord injury[60]. |
| Adam12 | 2.628 | Contributes to TGF-β signalling[61]. Secreted form promotes myogenesis[62], while anchored form interferes with re-epithelialization[63]. |
| Fbn2 | 2.557 | Found in wounds[64]. Needed for proper activation of latent TGF-β[61]. Sequesters BMP complexes in a latent state[65,66]. |
| Wisp1 | 2.546 | Role in wound healing suggested[11]. Inhibits SMAD2 mediated TGF-β1 signalling in osteoblasts[67]. |
| Inhba | 2.477 | Subunit of activin – a crucial factor in wound healing[12] Expressed in the granulation tissue[13]. |
| Aspn | 2.476 | Found after spinal cord injury[14] Inhibits TGF-β/SMAD signalling in osteoarthritis settings[15,16]. |
| Ltbp2 | 2.092 | May bind and modulate FGF-2 effects in wound repair and contribute to fibrotic diseases[28]. Does not bind to latent TGF-β, but might indirectly regulate the activation of TGF-β by releasing LTBP-1 from microfibrils[68,69]. |
| Dab2 | 1.871 | Dab2 attenuates brain injury by targeting TGF-β signalling pathway[70] and modulates TGF-β function in cancer[71]. |
| Loxl2 | 1.716 | Loxl2 promotes TGF-β signalling and is implicated in tissue fibrosis and fibroblast-to-myofibroblast conversion by inducing TGF-β2 secretion[72,73]. |
| Plod1 | 1.697 | Generates hydroxylysine residues from lysine residues in collagen peptides[74]. Mechanical state of the matrix can directly control the efficacy of TGF-β activation[75]. |
| Cilp | 1.638 | Inhibition of TGF-β1 signalling[76]. TGF-β1 induces CILP suggesting functional feedback loop[77]. |
| Lox | 1.606 | Profibrotic – increases ECM stiffness[78]. Binds to mature TGF-β1 and enzymatically regulates its signalling in the bone[79]. |
| Tgfbi | 1.455 | Is induced by TGF-β and plays role in wound healing[80]. Inhibition of TGFBIp expression leads to reduced myofibroblast differentiation[81]. |
| Bmp1 | 1.431 | BMP1-like proteinases are essential to the wound healing of skin[82] BMP1 controls TGF-β1 activation[83]. |
| Fstl1 | 1.369 | Failure of wound closure in the absence of Follistatin1[84]. Binds BMP4[85]. |
| Fbn1 | 1.271 | Fbn1 regulates TGF-β1 bioavailability[86]. |

| | | |
|---|---|---|
| Plod2 | 1.258 | Important enzyme in fibrosis[87]. Expression is regulated by an alternative downstream TGF-β1 activation mechanism[88]. Regulates ECM stiffness – myofibroblast differentiation[89]. |

**Supplementary Table 3** Top upregulated chemotaxis and inflammation related genes with reported functional relevance.

| Gene name | Log2 FC | Reported function |
|---|---|---|
| Cxcl5 | 3.653 | A potent neutrophil chemoattractant during allergic airway inflammation[90]. |
| Il1rl1 | 3.461 | The resulting soluble and receptor proteins have emerged as key regulators of the inflammatory process implicated in a large variety of human pathologies[91]. |
| Trem2 | 3.048 | TREM1 and TREM2 activate myeloid cells by signalling through the adaptor protein DAP12[92]. |
| Tnfsf14 | 2.309 | LIGHT plays role in the resolution of inflammation in wound healing[17], promotes collagen deposition and epidermal thickening and fibrosis in mouse skin[18]. It has been shown to be upregulated at the site of regeneration following nerve injury, but not reported in Schwann cells[19,20]. |
| Pla2g7 | 2.229 | PAF acetylhydrolase has the substrate specificity and lipoprotein association of the native enzyme, and blocks inflammation in vivo[93]. |
| Ccl8 | 1.700 | Monocyte chemotactic protein-2 activates CCR5[94]. |
| Cfp | 1.648 | Properdin plays a key role in allergen-induced airway inflammation and represents a potential therapeutic target for human asthma[95]. |
| Cd36 | 1.450 | A scavenger receptor involved in immunity, metabolism, angiogenesis, and behavior[96]. |
| Cxcl2 | 1.417 | It promotes the recruitment of myeloid-derived suppressor cells[97]. |

**Supplementary Table 4** Top upregulated migration and adhesion related genes with reported functional relevance.

| Gene name | Log2 FC | Reported function |
|---|---|---|
| Postn | 3.044 | Activates TGF-β pathway[54]. Promotes wound healing[55,56] and myofibroblast differentiation in the wound[57]. Important for wound melanoma metastasis[58] and Schwann cell migration[59]. Astroglial periostin promotes axonal regeneration after spinal cord injury [60]. |
| Col8a1 | 2.817 | Necessary for migration and proliferation of vascular smooth muscle cells and thus, has a potential role in the maintenance of vessel wall integrity and structure, in particular in atherogenesis[98]. |
| Adam12 | 2.628 | Anchored form interferes with re-epithelialization[62]. Marker that identifies a distinct pro-inflammatory subset of platelet-derived growth factor receptor-α-positive stromal cells that are activated upon acute injury in the muscle and dermis[99]. |
| Emcn | 2.561 | Interferes with the assembly of focal adhesion complexes and inhibits interaction between cells and the extracellular matrix[100]. |
| Thbs4 | 2.299 | Role for the astrocyte-secreted protein Thbs4 in the migration of newly formed neurons within the RMS to the olfactory bulb[101]. |
| Cdh11 | 2.225 | Regulates collagen and elastin synthesis, both affecting the mechanical properties and contractile function of animal tissues[102]. |
| Ccl6 | 2.224 | Also known as MIP-1, it is abundant in acute wounds and serves as macrophage chemoattractant[103,104]. Leads to CCR1 mediated signalling[105]. |
| Mfap4 | 2.125 | May contribute to the elastic fibre assembly and/or maintenance[106]. |
| Thbs2 | 1.922 | Adhesive glycoprotein that mediates cell-to-cell and cell-to-matrix interactions. Ligand for CD36 mediating antiangiogenic properties[107]. |
| Ccdc80 | 1.764 | Promotes cell adhesion and matrix assembly[108]. |
| Fbln2 | 1.687 | Acts as an adapter that mediates the interaction between FBN1 and ELN[109]. |
| Mfap5 | 1.216 | Protein associated with elastic fibre microfibrils[110]. |
| Spp1 | 1.179 | Binds tightly to hydroxyapatite. Appears to form an integral part of the mineralized matrix[111]. |

**Supplementary Table 5** Top upregulated ECM related genes with reported functional relevance.

| Gene name | Log2 FC | Reported function |
|---|---|---|
| Apln | 3.778 | Ectopic expression in keratinocytes induces enlarged but not leaky blood vessels in the dermis[112]. |
| Col8a1 | 2.817 | Necessary for migration and proliferation of vascular smooth muscle cells and thus, has a potential role in the maintenance of vessel wall integrity and structure, in particular in atherogenesis[98]. |
| Adam12 | 2.628 | Marker that identifies a distinct pro-inflammatory subset of platelet-derived growth factor receptor-α-positive stromal cells that are activated upon acute injury in the muscle and dermis[99]. |
| Col1a1 | 2.616 | Mutation in the gene cause Ehlers-Danlos syndrome, which is a connective tissue disorder characterized by hyperextensible skin, atrophic cutaneous scars due to tissue fragility and joint hyperlaxity[113]. Structural element of Type I Collagen, an ECM component important for wound matrix integrity at different stages of tissue repair[114]. |
| Emcn | 2.561 | Interferes with the assembly of focal adhesion complexes and inhibits interaction between cells and the extracellular matrix[100] |
| Fbn2 | 2.557 | Found in wounds[64]. Needed for proper activation of latent TGF-β[65]. Sequesters BMP complexes in a latent state[65,66]. |
| Col3a1 | 2.543 | Mutation in the gene cause Ehlers-Danlos syndrome 3 which is a connective tissue disorder characterized by hyperextensible skin, atrophic cutaneous scars due to tissue fragility and joint hyperlaxity. Is the major ligand of ADGRG1 in the developing brain and binding to ADGRG1 inhibits neuronal migration and activates the RhoA pathway by coupling ADGRG1 to GNA13 and possibly GNA12[115]. |
| Eln | 2.369 | Essential determinant of arterial morphogenesis. Possess regulatory function during arterial development, controlling proliferation of smooth muscle and stabilizing arterial structure[116]. |
| Col1a2 | 2.345 | Structural element of Type I Collagen, an ECM component important for wound matrix integrity at different stages of tissue repair[114]. Loss of Col1a2 can cause Ehlers-Danlos syndrome[117]. |
| Col5a2 | 2.343 | Mutation in the gene cause Ehlers-Danlos syndrome classic type which is a connective tissue disorder characterized by hyperextensible skin, atrophic cutaneous scars due to tissue fragility and joint hyperlaxity[118]. |
| Col6a5 | 2.090 | Mutations in the gene reported to be associated with atopic dermatitis using a family-based association analysis[119]. |
| Col5a1 | 2.020 | Mutation in the gene cause Ehlers-Danlos syndrome classic type which is a connective tissue disorder characterized by hyperextensible skin, atrophic cutaneous scars due to tissue fragility and joint hyperlaxity[118]. |
| Col6a1 | 1.964 | COL6A1 may serve participate in the initiation and progression of cervical cancer. It correlates with poor prognosis in cervical cancer patients[120]. |

| | | |
|---|---|---|
| Serpine1 | 1.864 | May function as a major control point in the regulation of fibrinolysis[121]. |

**Supplementary Table 6** Top upregulated angiogenesis related genes with reported functional relevance.

| Gene name | Log2 FC | Reported function |
|---|---|---|
| Esm1 | 4.015 | May play role in new blood vessel formation, affects VEGF bioavailability[122]. |
| Cxcl5 | 3.653 | CXCL5 is required for increased intestinal angiogenesis during resection-induced adaptation[123]. |
| Vash1 | 2.369 | Vash1 inhibits neo-vascularization upon injury[124]. |
| Sfrp2 | 2.302 | Sfrp2 inhibits apoptosis in Hypertrophic scars[125]. |
| Robo4 | 1.933 | SLIT3/ROBO4 pathway is required for MSC-guided vascularization in engineered tissues[126]. |
| Mdk | 1.751 | Mdk is associated with PGRN and promotes hepatocellular carcinoma angiogenesis[127]. Astrocyte secreted Mdk promotes CNS repair after injury[128]. |
| Hgf | 1.746 | Enhances angiogenesis in skin wounds[41]. Scatter factor (hepatocyte growth factor) is a potent angiogenesis factor *in vivo*[129]. |
| Fbln2 | 1.687 | FBLN2 isoform has an important tumour-suppressive and anti-angiogenic role in nasopharyngeal carcinoma[130]. |
| Vcan | 1.678 | Versican V2 isoform enhances angiogenesis by regulating endothelial cell activities[131]. |

**Supplementary Table 7** Genotyping primers.

| Allele | Forward sequence | Reverse sequence |
|---|---|---|
| *Cre* | CTATCCAGCAACATTTGGGCCAGC | CCAGGTTACGGATATAGTTCATGAC |
| *$Pten^{lox}$* | CAAGCACTCTGCGAACTGAG | AAGTTTTTGAAGGCAAGATGC |
| *Rosa-LacZ* | GGTCGGCTTACGGCGGTGATTT | AGCGGCGTCAGCAGTTGTTTTT |
| *$Sox10^{lox}WT$* | CAGGTGGGCGTTGGGCTCTT | TCCCAGGCTAGCCCTAGTG |
| *$Sox10^{lox}pos$* | | GTGAGCCTGGATAGCAGCAG |
| *TdTomato null* | AAGGGAGCTGCAGTGGAGTA | CCGAAAATCTGTGGGAAGTC |
| *TdTomato pos* | CTGTTCCTGTACGGCATGG | GGCATTAAAGCAGCGTATCC |

**Supplementary Table 8** Primary antibodies.

| Epitope | Reactivity | Producer | Catalog Nr. | Dilution |
|---|---|---|---|---|
| β-actin | human / mouse | Sigma | A5316 | WB, 1:5000 |
| CD271/p75 | human / mouse | Alomone Labs | ANT-007 | IF, 1:200 |
| CD31 | human / mouse | Abcam | ab28364 | IF, 1:200 |
| CD45 | mouse | BD Pharmingen | 550539 | IF, 1:200 |
| C-JUN | human / mouse | SC Biotechnology | SC-1694 | IF, 1:200 |
| DCT | human / mouse | SC Biotechnology | SC-10451 | IF, 1:250 |
| GFAP | human / mouse | Dako | Z0034 | IF, 1:300 |
| KI67 | mouse | BioLegend | 652402 | IF, 1:100 |
| LAMININ | human / mouse | Sigma | L9393 | IF, 1:200 |
| MBP | human / mouse | Abd Serotec | aa82-87 | IF, 1:300 |
| Neurofilament-M | human / mouse | EMD Millipore | AB1987 | IF, 1:300 |
| pAKT | human / mouse | Cell Signalling | 4058 | IF, 1:200 |
| pERK1/2 | human / mouse | Cell Signalling | 9101 | IF, 1:300 |
| Periostin | human / mouse | SC Biotechnology | SC-49480 | IF, 1:200<br>WB 1:1000 |
| pSMAD2 (138D4) | human / mouse | Cell Signalling | 3108 | IF, 1:300 |
| RFP | NA | EMD Millipore | AB3216 | IF, 1:100 |
| α-SMA | human / mouse | Sigma | A2547 | IF, 1:100 |
| α-SMA | mouse/rat | Abcam | ab5694 | WB, 1:1000 |
| SOX10 | human / mouse | SC Biotechnology | SC-17342 | IF, 1:100 |
| TdTomato | NA | LS Bio | LS-C340696 | IF, 1:200 |

**Supplementary Table 9** Secondary antibodies.

| **Secondary antibody** | **Producer** | **Catalog Nr.** |
|---|---|---|
| Cy3 Donkey anti-goat IgG (H+L) | Jackson | 705-165-147 |
| Alexa Fluor 488-conjugated AffiniPure Donkey anti-goat IgG (H+L) | Jackson | 705-545-147 |
| Cy3 Donkey anti-rabbit IgG (H+L) | Jackson | 711-165-152 |
| Alexa Fluor® 488-AffiniPure Donkey Anti-Rabbit IgG (H+L) | Jackson | 711-545-152 |
| Alexa Fluor 647-conjugated Affin Pure Donkey anti mouse IgG | Jackson | 715-605-150 |
| Alexa Fluor® 647-AffiniPure Donkey Anti-Rat IgG (H+L) | Jackson | 712-605-153 |
| Biotin-SP Donkey anti-rabbit IgG (H&L) | Jackson | 711-065-152 |
| HRP Streptavidin | Jackson | 016-030-084 |
| IRDye 800 CW Donkey anti-goat | Li-Cor | 926-32214 |
| IRDye 800 CW Donkey anti-mouse | Li-Cor | 926-32212 |
| RDye 680 LT Donkey anti-rabbit | Li-Cor | 926-68022 |

## Supplementary References


1. Neidhardt, J., Fehr, S., Kutsche, M., Löhler, J. & Schachner, M. Tenascin-N: Characterization of a novel member of the tenascin family that mediates neurite repulsion from hippocampal explants. *Mol. Cell. Neurosci.* **23,** 193–209 (2003).
2. Meloty-Kapella, C. V., Degen, M., Chiquet-Ehrismann, R. & Tucker, R. P. Avian tenascin-W: Expression in smooth muscle and bone, and effects on calvarial cell spreading and adhesion in vitro. *Dev. Dyn.* **235,** 1532–1542 (2006).
3. Brellier, F. *et al.* The adhesion modulating properties of tenascin-W. *Int. J. Biol. Sci.* **8,** 187–194 (2011).
4. Stoll, S. W. *et al.* The EGF receptor ligand amphiregulin controls cell division via FoxM1. *Oncogene* 1–12 (2015). doi:10.1038/onc.2015.269
5. Schelfhout, V. R. *et al.* The role of heregulin-alpha as a motility factor and amphiregulin as a growth factor in wound healing. *J. Pathol.* **198,** 523–533 (2002).
6. Zhou, Y. *et al.* Amphiregulin, an epidermal growth factor receptor ligand, plays an essential role in the pathogenesis of transforming growth factor-β-induced pulmonary fibrosis. *J. Biol. Chem.* **287,** 41991–2000 (2012).
7. Zaiss, D. M. W., Gause, W. C., Osborne, L. C. & Artis, D. Emerging functions of amphiregulin in orchestrating immunity, inflammation, and tissue repair. *Immunity* **42,** 216–226 (2015).
8. Sumioka, T. *et al.* Impaired cornea wound healing in a tenascin C-deficient mouse model. *Lab. Invest.* **93,** 207–17 (2013).
9. Tamaoki, M. *et al.* Tenascin-C Regulates Recruitment of Myofibroblasts during Tissue Repair after Myocardial Injury. *Am. J. Pathol.* **167,** 71–80 (2005).
10. Fruttiger, M., Schachner, M. & Martini, R. Tenascin-C expression during Wallerian degeneration in C57BL/Wlds mice: possible implications for axonal regeneration. *J. Neurocytol.* **24,** 1–14 (1995).
11. Berschneider, B. & Königshoff, M. WNT1 inducible signaling pathway protein 1 (WISP1): A novel mediator linking development and disease. *Int. J. Biochem. Cell Biol.* **43,** 306–309 (2011).
12. Antsiferova, M. & Werner, S. The bright and the dark sides of activin in wound healing and cancer. *J. Cell Sci.* **125,** 3929–37 (2012).
13. Hübner, G., Hu, Q., Smola, H. & Werner, S. Strong Induction of Activin Expression after Injury Suggests an Important Role of Activin in Wound Repair. *Dev. Biol.* **173,** 490–498 (1996).
14. Didangelos, A. *et al.* High-throughput proteomics reveal alarmins as amplifiers of tissue pathology and inflammation after spinal cord injury. *Sci. Rep.* **6,** 21607 (2016).
15. Kizawa, H. *et al.* An aspartic acid repeat polymorphism in asporin inhibits chondrogenesis and increases susceptibility to osteoarthritis. *Nat. Genet.* **37,** 138–44 (2005).
16. Nakajima, M. *et al.* Mechanisms for asporin function and regulation in articular cartilage. *J. Biol. Chem.* **282,** 32185–32192 (2007).
17. Petreaca, M. L., Yao, M., Ware, C. & Martins-Green, M. M. Vascular endothelial growth factor promotes macrophage apoptosis through stimulation of tumor necrosis factor superfamily member 14 (TNFSF14/LIGHT). *Wound*

*Repair Regen.* **16,** 602–614 (2008).

18. Herro, R., Antunes, R. D. S., Aguilera, A. R., Tamada, K. & Croft, M. The Tumor Necrosis Factor Superfamily Molecule LIGHT Promotes Keratinocyte Activity and Skin Fibrosis. *J. Invest. Dermatol.* **135,** 2109–18 (2015).
19. Otsmane, B. *et al.* Somatic and axonal LIGHT signaling elicit degenerative and regenerative responses in motoneurons, respectively. *EMBO Rep.* **15,** 540–547 (2014).
20. Han, D. *et al.* Profiling of the dynamically alteredgene expression in peripheral nerve injury using NGS RNA sequencing technique. *Am. J. Transl. Res.* **8,** 871–84 (2016).
21. Schneider, M. R. *et al.* Betacellulin regulates hair follicle development and hair cycle induction and enhances angiogenesis in wounded skin. *J. Invest. Dermatol.* **128,** 1256–1265 (2008).
22. Byer, S. J. *et al.* Malignant peripheral nerve sheath tumor invasion requires aberrantly expressed EGF receptors and is variably enhanced by multiple EGF family ligands. *J. Neuropathol. Exp. Neurol.* **72,** 219–33 (2013).
23. Werner, S. & Grose, R. Regulation of wound healing by growth factors and cytokines. *Physiol. Rev.* **83,** 835–870 (2003).
24. Seo, H.-R. *et al.* Intrinsic FGF2 and FGF5 promotes angiogenesis of human aortic endothelial cells in 3D microfluidic angiogenesis system. *Sci. Rep.* **6,** 28832 (2016).
25. Hébert, J. M., Rosenquist, T., Götz, J. & Martin, G. R. FGF5 as a regulator of the hair growth cycle: Evidence from targeted and spontaneous mutations. *Cell* **78,** 1017–1025 (1994).
26. Barrette, B., Calvo, E., Vallières, N. & Lacroix, S. Transcriptional profiling of the injured sciatic nerve of mice carrying the Wld(S) mutant gene: identification of genes involved in neuroprotection, neuroinflammation, and nerve regeneration. *Brain. Behav. Immun.* **24,** 1254–67 (2010).
27. Irizarry-Ramírez, M. *et al.* Upregulation of EphA3 receptor after spinal cord injury. *J. Neurotrauma* **22,** 929–35 (2005).
28. Sideek, M. A., Teia, A., Kopecki, Z., Cowin, A. J. & Gibson, M. A. Co-localization of LTBP-2 with FGF-2 in fibrotic human keloid and hypertrophic scar. *J. Mol. Histol.* **47,** 35–45 (2016).
29. Gimm, T. *et al.* Hypoxia-inducible protein 2 is a novel lipid droplet protein and a specific target gene of hypoxia-inducible factor-1. *FASEB J.* **24,** 4443–4458 (2010).
30. Hong, W. X. *et al.* The Role of Hypoxia-Inducible Factor in Wound Healing. *Adv. wound care* **3,** 390–399 (2014).
31. Levy, A. *et al.* CD38 facilitates recovery from traumatic brain injury. *J. Neurotrauma* **26,** 1521–33 (2009).
32. Moeller, C., Swindell, E. C., Kispert, A. & Eichele, G. Carboxypeptidase Z (CPZ) modulates Wnt signaling and regulates the development of skeletal elements in the chicken. *Development* **130,** 5103–5111 (2003).
33. Mescher, A. L., Connell, E., Hsu, C., Patel, C. & Overton, B. Transferrin is necessary and sufficient for the neural effect on growth in amphibian limb regeneration blastemas. *Development, growth & differentiation* **39,** 677–84 (1997).
34. Salis, C., Setton, C. P., Soto, E. F. & Pasquini, J. M. The mRNA of transferrin

is expressed in Schwann cells during their maturation and after nerve injury. *Exp. Neurol.* **207,** 85–94 (2007).

35. Lynch, S. E., Nixon, J. C., Colvin, R. B. & Antoniades, H. N. Role of platelet-derived growth factor in wound healing: synergistic effects with other growth factors. *Proc. Natl. Acad. Sci. U. S. A.* **84,** 7696–7700 (1987).

36. Ehrlich, H. P. & Freedman, B. M. Topical platelet-derived growth factor in patients enhances wound closure in the absence of wound contraction. *Cytokines. Cell. Mol. Ther.* **7,** 85–90 (2002).

37. Park, S. A. *et al.* PDGF-BB does not accelerate healing in diabetic mice with splinted skin wounds. *PLoS One* **9,** (2014).

38. Nishino, J. *et al.* Meteorin: a secreted protein that regulates glial cell differentiation and promotes axonal extension. *EMBO J.* **23,** 1998–2008 (2004).

39. Wright, J. L., Ermine, C. M., Jørgensen, J. R., Parish, C. L. & Thompson, L. H. Over-Expression of Meteorin Drives Gliogenesis Following Striatal Injury. *Front. Cell. Neurosci.* **10,** 160–168 (2016).

40. Tufvesson, E. & Westergren-Thorsson, G. Biglycan and decorin induce morphological and cytoskeletal changes involving signalling by the small GTPases RhoA and Rac1 resulting in lung fibroblast migration. *J. Cell Sci.* **116,** 4857–4864 (2003).

41. Bevan, D., Gherardi, E., Fan, T. P., Edwards, D. & Warn, R. Diverse and potent activities of HGF/SF in skin wound repair. *J. Pathol.* **203,** 831–838 (2004).

42. Chmielowiec, J. *et al.* c-Met is essential for wound healing in the skin. *J. Cell Biol.* **177,** 151–162 (2007).

43. Li, J. F. *et al.* HGF accelerates wound healing by promoting the dedifferentiation of epidermal cells through ?? 1 -Integrin/ILK pathway. *Biomed Res. Int.* **2013,** (2013).

44. Maina, F. & Klein, R. Hepatocyte growth factor, a versatile signal for developing neurons. *Nat. Neurosci.* **2,** 213–217 (1999).

45. Hashimoto, N. *et al.* Expression of HGF and cMet in the peripheral nervous system of adult rats following sciatic nerve injury. *Neuroreport* **12,** 1403–7 (2001).

46. Ma, L., Shen, Y.-Q., Khatri, H. P. & Schachner, M. The Asparaginyl Endopeptidase Legumain Is Essential for Functional Recovery after Spinal Cord Injury in Adult Zebrafish. *PLoS One* **9,** e95098 (2014).

47. Jafari, A. *et al.* Legumain Regulates Differentiation Fate of Human Bone Marrow Stromal Cells and Is Altered in Postmenopausal Osteoporosis. *Stem Cell Reports* **8,** (2017).

48. Hase, A. *et al.* Characterization of glial cell line-derived neurotrophic factor family receptor alpha-1 in peripheral nerve Schwann cells. *J. Neurochem.* **95,** 537–43 (2005).

49. Honma, M. *et al.* Developmental alteration of nerve injury induced glial cell line-derived neurotrophic factor (GDNF) receptor expression is crucial for the determination of injured motoneuron fate. *J. Neurochem.* **82,** 961–975 (2002).

50. Haase, I., Evans, R., Pofahl, R. & Watt, F. M. Regulation of keratinocyte shape, migration and wound epithelialization by IGF-1- and EGF-dependent signalling pathways. *J. Cell Sci.* **116,** 3227–3238 (2003).

51. Emmerson, E. *et al.* Insulin-like growth factor-1 promotes wound healing in estrogen-deprived mice: new insights into cutaneous IGF-1R/ERα cross talk. *J. Invest. Dermatol.* **132,** 2838–48 (2012).

52. Hansson, H. A. *et al.* Evidence indicating trophic importance of IGF-I in regenerating peripheral nerves. *Acta Physiol. Scand.* **126,** 609–14 (1986).

53. Apel, P. J. *et al.* Effect of locally delivered IGF-1 on nerve regeneration during aging: An experimental study in rats. *Muscle and Nerve* **41,** 335–341 (2010).

54. Sidhu, S. S. *et al.* Roles of epithelial cell-derived periostin in TGF-beta activation, collagen production, and collagen gel elasticity in asthma. *Proc. Natl. Acad. Sci. U. S. A.* **107,** 14170–14175 (2010).

55. Ontsuka, K. *et al.* Periostin, a matricellular protein, accelerates cutaneous wound repair by activating dermal fibroblasts. *Exp. Dermatol.* **21,** 331–336 (2012).

56. Nishiyama, T. *et al.* Delayed re-epithelialization in periostin-deficient mice during cutaneous wound healing. *PLoS One* **6,** (2011).

57. Elliott, C. G. *et al.* Periostin modulates myofibroblast differentiation during full-thickness cutaneous wound repair. *J. Cell Sci.* **125,** 121–32 (2012).

58. Fukuda, K. *et al.* Periostin Is a Key Niche Component for Wound Metastasis of Melanoma. *Plos One* **10,** e0129704 (2015).

59. Sonnenberg-Riethmacher, E., Miehe, M. & Riethmacher, D. Promotion of periostin expression contributes to the migration of Schwann cells. *J. Cell Sci.* **128,** 3345–3355 (2015).

60. Shih, C.-H., Lacagnina, M., Leuer-Bisciotti, K. & Proschel, C. Astroglial-Derived Periostin Promotes Axonal Regeneration after Spinal Cord Injury. *J. Neurosci.* **34,** 2438–2443 (2014).

61. Atfi, A. *et al.* The disintegrin and metalloproteinase ADAM12 contributes to TGF-β signaling through interaction with the type II receptor. *J. Cell Biol.* **178,** 201–208 (2007).

62. Gilpin, B. J. *et al.* A novel secreted form of human ADAM 12 (meltrin α) provokes myogenesis in vivo. *J. Biol. Chem.* **273,** 157–166 (1998).

63. Harsha, A. *et al.* ADAM12: a potential target for the treatment of chronic wounds. *J. Mol. Med. (Berl).* **86,** 961–9 (2008).

64. Brinckmann, J. *et al.* Enhanced fibrillin-2 expression is a general feature of wound healing and sclerosis: potential alteration of cell attachment and storage of TGF-beta. *Lab. Invest.* **90,** 739–752 (2010).

65. Nistala, H. *et al.* Fibrillin-1 and -2 differentially modulate endogenous TGF-β and BMP bioavailability during bone formation. *J. Cell Biol.* **190,** 1107–1121 (2010).

66. Sengle, G. *et al.* Abnormal Activation of BMP Signaling Causes Myopathy in Fbn2 Null Mice. *PLoS Genet.* **11,** (2015).

67. Inkson, C. A. *et al.* TGF-β1 and WISP-1/CCN-4 can regulate each other's activity to cooperatively control osteoblast function. *J. Cell. Biochem.* **104,** 1865–1878 (2008).

68. Robertson, I. B. *et al.* Latent TGF-β-binding proteins. *Matrix Biol.* **47,** 44–53 (2015).

69. Hirani, R., Hanssen, E. & Gibson, M. A. LTBP-2 specifically interacts with the amino-terminal region of fibrillin-1 and competes with LTBP-1 for binding to this microfibrillar protein. *Matrix Biol.* **26,** 213–223 (2007).

70. Song, L. *et al.* Dab2 attenuates brain injury in APP/PS1 mice via targeting transforming growth factor-beta/SMAD signaling. *Neural Regen. Res.* **9,** 41–50 (2014).

71. Hannigan, A. *et al.* Epigenetic downregulation of human disabled homolog 2 switches TGF-β from a tumor suppressor to a tumor promoter. *J. Clin. Invest.* **120,** 2842–2857 (2010).

72. Barry-Hamilton, V. *et al.* Allosteric inhibition of lysyl oxidase-like-2 impedes the development of a pathologic microenvironment. *Nat. Med.* **16,** 1009–17 (2010).

73. Yang, J. *et al.* Targeting LOXL2 for cardiac interstitial fibrosis and heart failure treatment. *Nat. Commun.* **7,** 13710 (2016).

74. Witsch, T. J. *et al.* Deregulation of the lysyl hydroxylase matrix cross-linking system in experimental and clinical bronchopulmonary dysplasia. *Am. J. Physiol. Lung Cell. Mol. Physiol.* **306,** L246-59 (2014).

75. Hinz, B. The extracellular matrix and transforming growth factor-β1: Tale of a strained relationship. *Matrix Biology* **47,** 54–65 (2015).

76. Seki, S. *et al.* A functional SNP in CILP, encoding cartilage intermediate layer protein, is associated with susceptibility to lumbar disc disease. *Nat. Genet.* **37,** 607–612 (2005).

77. Mori, M. *et al.* Transcriptional regulation of the cartilage intermediate layer protein (CILP) gene. *Biochem. Biophys. Res. Commun.* **341,** 121–127 (2006).

78. Levental, K. R. *et al.* Matrix Crosslinking Forces Tumor Progression by Enhancing Integrin Signaling. *Cell* **139,** 891–906 (2009).

79. Atsawasuwan, P. *et al.* Lysyl oxidase binds transforming growth factor-beta and regulates its signaling via amine oxidase activity. *J Biol Chem* **283,** 34229–34240 (2008).

80. Thapa, N., Lee, B. H. & Kim, I. S. TGFBIp/βig-h3 protein: A versatile matrix molecule induced by TGF-β. *International Journal of Biochemistry and Cell Biology* **39,** 2183–2194 (2007).

81. Kim, T.-I. *et al.* Inhibitory Effect of Tranilast on Transforming Growth Factor-Beta-Induced Protein in Granular Corneal Dystrophy Type 2 Corneal Fibroblasts. *Cornea* **34,** 950–958 (2015).

82. Muir, A. M. *et al.* BMP1-like proteinases are essential to the structure and wound healing of skin. *Matrix Biol.* **276,** 75–81 (2016).

83. Ge, G. & Greenspan, D. S. BMP1 controls TGFbeta1 activation via cleavage of latent TGFbeta-binding protein. *J. Cell Biol.* **175,** 111–20 (2006).

84. Sundaram, G. M. *et al.* 'See-saw' expression of microRNA-198 and FSTL1 from a single transcript in wound healing. *Nature* **495,** 103–6 (2013).

85. Geng, Y. *et al.* Follistatin-like 1 (Fstl1) is a bone morphogenetic protein (BMP) 4 signaling antagonist in controlling mouse lung development. *Proc. Natl. Acad. Sci.* **108,** 7058–7063 (2011).

86. Chaudhry, S. S. *et al.* Fibrillin-1 regulates the bioavailability of TGFβ1. *J. Cell Biol.* **176,** 355–367 (2007).

87. Van der Slot, A. J. *et al.* Identification of PLOD2 as Telopeptide Lysyl Hydroxylase, an Important Enzyme in Fibrosis. *J. Biol. Chem.* **278,** 40967–40972 (2003).

88. Gjaltema, R. A. F., De Rond, S., Rots, M. G. & Bank, R. A. Procollagen lysyl hydroxylase 2 expression is regulated by an alternative downstream

transforming growth factor β-1 activation mechanism. *J. Biol. Chem.* **290,** 28465–28476 (2015).

89. Hinz, B. *et al.* Recent developments in myofibroblast biology: Paradigms for connective tissue remodeling. *American Journal of Pathology* **180,** 1340–1355 (2012).

90. Lukacs, N. W. *et al.* Mast cells produce ENA-78, which can function as a potent neutrophil chemoattractant during allergic airway inflammation. *J. Leukoc. Biol.* **63,** 746–51 (1998).

91. Akhabir, L. & Sandford, A. Genetics of interleukin 1 receptor-like 1 in immune and inflammatory diseases. *Curr. Genomics* **11,** 591–606 (2010).

92. Colonna, M. TREMs in the immune system and beyond. *Nat. Rev. Immunol.* **3,** 445–53 (2003).

93. Tjoelker, L. W. *et al.* Anti-inflammatory properties of a platelet-activating factor acetylhydrolase. *Nature* **374,** 549–53 (1995).

94. Gong, W. *et al.* Monocyte chemotactic protein-2 activates CCR5 and blocks CD4/CCR5-mediated HIV-1 entry/replication. *J. Biol. Chem.* **273,** 4289–92 (1998).

95. Wang, Y. *et al.* Properdin Contributes to Allergic Airway Inflammation through Local C3a Generation. *J. Immunol.* **195,** 1171–1181 (2015).

96. Silverstein, R. L. & Febbraio, M. CD36, a scavenger receptor involved in immunity, metabolism, angiogenesis, and behavior. *Sci. Signal.* **2,** re3 (2009).

97. Zhang, H. *et al.* CXCL2/MIF-CXCR2 signaling promotes the recruitment of myeloid-derived suppressor cells and is correlated with prognosis in bladder cancer. *Oncogene* 1–10 (2016). doi:10.1038/onc.2016.367

98. Xu, R. *et al.* NC1 domain of human type VIII collagen (alpha 1) inhibits bovine aortic endothelial cell proliferation and causes cell apoptosis. *Biochem. Biophys. Res. Commun.* **289,** 264–8 (2001).

99. Dulauroy, S., Di Carlo, S. E., Langa, F., Eberl, G. & Peduto, L. Lineage tracing and genetic ablation of ADAM12+ perivascular cells identify a major source of profibrotic cells during acute tissue injury. *Nat. Med.* **18,** 1262–1270 (2012).

100. Kinoshita, M. *et al.* Identification of human endomucin-1 and -2 as membrane-bound O-sialoglycoproteins with anti-adhesive activity. *FEBS Lett.* **499,** 121–6 (2001).

101. Girard, F., Eichenberger, S. & Celio, M. R. Thrombospondin 4 deficiency in mouse impairs neuronal migration in the early postnatal and adult brain. *Mol. Cell. Neurosci.* **61,** 176–86 (2014).

102. Row, S., Liu, Y., Alimperti, S., Agarwal, S. K. & Andreadis, S. T. Cadherin-11 is a novel regulator of extracellular matrix synthesis and tissue mechanics. *J. Cell Sci.* **129,** 2950–61 (2016).

103. Low, Q. E. H. *et al.* Wound Healing in MIP-1α−/− and MCP-1−/− Mice. *Am. J. Pathol.* **159,** 457–463 (2001).

104. DiPietro, L. A., Burdick, M., Low, Q. E., Kunkel, S. L. & Strieter, R. M. Mip-1α as a critical macrophage chemoattractant in murine wound repair. *J. Clin. Invest.* **101,** 1693–1698 (1998).

105. Berahovich, R. D. *et al.* Proteolytic activation of alternative CCR1 ligands in inflammation. *J. Immunol.* **174,** 7341–51 (2005).

106. Pilecki, B. *et al.* Characterization of Microfibrillar-associated Protein 4 (MFAP4) as a Tropoelastin- and Fibrillin-binding Protein Involved in Elastic Fiber

Formation. *J. Biol. Chem.* **291,** 1103–14 (2016).

107. Koch, M. *et al.* CD36-mediated activation of endothelial cell apoptosis by an N-terminal recombinant fragment of thrombospondin-2 inhibits breast cancer growth and metastasis in vivo. *Breast Cancer Res. Treat.* **128,** 337–46 (2011).
108. Manabe, R. *et al.* Transcriptome-based systematic identification of extracellular matrix proteins. *Proc. Natl. Acad. Sci. U. S. A.* **105,** 12849–54 (2008).
109. El-Hallous, E. *et al.* Fibrillin-1 interactions with fibulins depend on the first hybrid domain and provide an adaptor function to tropoelastin. *J. Biol. Chem.* **282,** 8935–46 (2007).
110. Gibson, M. A. *et al.* Further characterization of proteins associated with elastic fiber microfibrils including the molecular cloning of MAGP-2 (MP25). *J. Biol. Chem.* **271,** 1096–103 (1996).
111. Hunter, C., Bond, J., Kuo, P. C., Selim, M. A. & Levinson, H. The role of osteopontin and osteopontin aptamer (OPN-R3) in fibroblast activity. *J. Surg. Res.* **176,** 348–58 (2012).
112. Kidoya, H., Naito, H. & Takakura, N. Apelin induces enlarged and nonleaky blood vessels for functional recovery from ischemia. *Blood* **115,** 3166–74 (2010).
113. Nuytinck, L. *et al.* Classical Ehlers-Danlos syndrome caused by a mutation in type I collagen. *Am. J. Hum. Genet.* **66,** 1398–1402 (2000).
114. Midwood, K. S., Williams, L. V. & Schwarzbauer, J. E. Tissue repair and the dynamics of the extracellular matrix. *International Journal of Biochemistry and Cell Biology* **36,** 1031–1037 (2004).
115. Luo, R. *et al.* G protein-coupled receptor 56 and collagen III, a receptor-ligand pair, regulates cortical development and lamination. *Proc. Natl. Acad. Sci. U. S. A.* **108,** 12925–30 (2011).
116. Li, D. Y. *et al.* Elastin is an essential determinant of arterial morphogenesis. *Nature* **393,** 276–80 (1998).
117. Malfait, F. *et al.* Total absence of the alpha2(I) chain of collagen type I causes a rare form of Ehlers-Danlos syndrome with hypermobility and propensity to cardiac valvular problems. *J. Med. Genet.* **43,** e36 (2006).
118. De Paepe, A., Nuytinck, L., Hausser, I., Anton-Lamprecht, I. & Naeyaert, J. M. Mutations in the COL5A1 gene are causal in the Ehlers-Danlos syndromes I and II. *Am. J. Hum. Genet.* **60,** 547–54 (1997).
119. Söderhäll, C. *et al.* Variants in a novel epidermal collagen gene (COL29A1) are associated with atopic dermatitis. *PLoS Biol.* **5,** e242 (2007).
120. Hou, T. *et al.* Expression of COL6A1 predicts prognosis in cervical cancer patients. *Am. J. Transl. Res.* **8,** 2838–44 (2016).
121. Szabo, R., Netzel-Arnett, S., Hobson, J. P., Antalis, T. M. & Bugge, T. H. Matriptase-3 is a novel phylogenetically preserved membrane-anchored serine protease with broad serpin reactivity. *Biochem. J.* **390,** 231–42 (2005).
122. Rocha, S. F. *et al.* Esm1 modulates endothelial tip cell behavior and vascular permeability by enhancing VEGF bioavailability. *Circ. Res.* **115,** 581–90 (2014).
123. Rowland, K. J. *et al.* CXCL5 is required for angiogenesis, but not structural adaptation after small bowel resection. *J. Pediatr. Surg.* **49,** 976–80; discussion 980 (2014).

124. Zhou, S. *et al.* Inhibition of mouse alkali burn induced-corneal neovascularization by recombinant adenovirus encoding human vasohibin-1. *Mol. Vis.* **16,** 1389–98 (2010).

125. Kaur, A. *et al.* sFRP2 in the aged microenvironment drives melanoma metastasis and therapy resistance. *Nature* 1–17 (2016). doi:10.1038/nature17392

126. Paul, J. D. *et al.* SLIT3-ROBO4 activation promotes vascular network formation in human engineered tissue and angiogenesis in vivo. *J. Mol. Cell. Cardiol.* **64,** 124–31 (2013).

127. Huang, H. *et al.* Role of midkine-progranulin interaction during angiogenesis of hepatocellular carcinoma. *Int. J. Clin. Exp. Pathol.* **8,** 8809–20 (2015).

128. Yoshida, Y., Sakakima, H., Matsuda, F. & Ikutomo, M. Midkine in repair of the injured nervous system. *British Journal of Pharmacology* **171,** 924–930 (2014).

129. Rosen, E. M. *et al.* Scatter factor (hepatocyte growth factor) is a potent angiogenesis factor in vivo. *Symp. Soc. Exp. Biol.* **47,** 227–34 (1993).

130. Law, E. W. L. *et al.* Anti-angiogenic and tumor-suppressive roles of candidate tumor-suppressor gene, Fibulin-2, in nasopharyngeal carcinoma. *Oncogene* **31,** 728–38 (2012).

131. Yang, W. & Yee, A. J. Versican V2 isoform enhances angiogenesis by regulating endothelial cell activities and fibronectin expression. *FEBS Lett.* **587,** 185–92 (2013).
